# Molecular yield of targeted sequencing for Glanzmann thrombasthenia patients

**DOI:** 10.1038/s41525-019-0079-6

**Published:** 2019-02-14

**Authors:** Tarek Owaidah, Mahasen Saleh, Batoul Baz, Basma Abdulaziz, Hazza Alzahrani, Ahmed Tarawah, Abdulrahman Almusa, Randa AlNounou, Hala AbaAlkhail, Nouf Al-Numair, Rahaf Altahan, Mohammed Abouelhoda, Thamer Alamoudi, Dorota Monies, Amjad Jabaan, Nada Al Tassan

**Affiliations:** 1 Department of Pathology and Laboratory Medicine, King Faisal Specialist Hospital and Research Centre, Riyadh, Saudi Arabia; 2 Saudi Human Genome Program, King Abdulaziz City for Science and Technology, Riyadh, Saudi Arabia; 3 Department of Genetics, King Faisal Specialist Hospital and Research Centre, Riyadh, Saudi Arabia; 4 Medina Maternity and Children Hospital, Medina, Saudi Arabia

## Abstract

Glanzmann thrombasthenia (GT) is a rare autosomal recessive bleeding disorder. Around 490 mutations in *ITGA2B* and *ITGB3* genes were reported. We aimed to use targeted next-generation sequencing (NGS) to identify variants in patients with GT. We screened 72 individuals (including unaffected family members) using a panel of 393 genes (SHGP heme panel). Validation was done by Sanger sequencing and pathogenicity was predicted using multiple tools. In 83.5% of our cohort, 17 mutations were identified in *ITGA2B* and *ITGB3* (including 6 that were not previously reported). In addition to variants in the two known genes, we found variants in *ITGA2*, *VWF* and *F8*. The SHGP heme panel can be used as a high-throughput molecular diagnostic assay to screen for mutations and variants in GT cases and carriers. Our findings expand the molecular landscape of GT and emphasize the robustness and usefulness of this panel.

## Introduction

Glanzmann thrombasthenia (GT) is a rare autosomal recessive bleeding disorder where spontaneous mucocutaneous bleeding and easy bruising are classical hemorrhagic symptoms. It is characterized by mutations in *Integrin Subunit Alpha 2b* (*ITGA2B*; Online Mendelian Inheritance in Man (OMIM) #607759) and *Integrin Subunit Beta 3* (*ITGB3;* OMIM #173470) genes that code for the alpha and beta subunits of the platelet membrane adhesive protein receptor complex GPIIb/IIIa (αIIbβ3), respectively.^1^ Mutations in these genes prevent expression and/or functioning of αIIbβ3 and affect platelet aggregation. A bleeding phenotype results from failure in forming platelet aggregates that are required to seal damaged vessel walls through the receptor’s interactions with its adhesive ligands: fibrinogen (Fg), Von Willebrand factor (VWF) and others.^1–3^

The clinical presentation and consequent bleeding complications observed clinically in patients diagnosed with GT vary widely, ranging from minimal or moderate to severe. Among the common features, patients present with purpura, epistaxis, gingival hemorrhages and menorrhagia. However, gastrointestinal bleeding and hematuria are less frequent, and so is unprovoked bleeding. Prolonged bleeding time and deficient or decreased clot retraction are common features, despite normal platelet count and morphology.^1–3^

There are three major types of GT clinical classifications. Patients presenting with less than 5% of normal αIIbβ3 expression are designated as type I. This type is considered the most severe form of the disease. The moderately deficient type is designated as type II, where patients show 10–20% of normal αIIbβ3 expression. Finally, patients with 50–100% of αIIbβ3 expression levels and a defective receptor function are classified as GT-variants.^1^

This hereditary disease is more common in populations where consanguineous marriages prevail.^4^ In other populations with comparable rates of consanguinity, GT has been reported among family members (Ashkenazi and Iraqi Jews, French Gypsy Manouche, Arabs and southern Indian communities). A review of 168 patients referred for bleeding disorders in a single hospital in Saudi Arabia found 18 of them (10.7%) to have GT.^5^ Studies involving Saudi patients diagnosed with GT found overlapping clinical features with mild von Willebrand’s disease^6^ and hemophilia.^7^

Mutations in both *ITGA2B* and *ITGB3* genes have been reported in GT patients, including truncating and missense single-nucleotide variants (SNVs), splice defects, in addition to deletions, insertions and inversions.^8^ Sequencing platelet RNA can be used for mutation screening. A limitation to this approach is the low expression and poor stability of RNA in platelets, keeping in mind that loss-of-function mutations themselves may affect RNA stability.^9^ A catalog of reported mutations in *ITGA2B* (30 exons) and *ITGB3* (15 exons) is accessible through an up-to-date database http://sinaicentral.mssm.edu/intranet/research/glanzmann.^10^ The database currently has a record of 236 mutations in *ITGA2B*, and 149 variants in *ITGB3*. The higher number of mutations in *ITGA2B* could be attributed to the large number of splice sites since this gene is made of 30 exons while *ITGB3* is made of 15 exons. The nature and position of the mutation determines the residual functional response.^11^

Many studies have been conducted to decipher genotype phenotype correlations in GT. Nurden et al.^8^ studied 76 affected GT families and identified 78 causal mutations by Sanger sequencing, including 55 novel variants. The study showed that patients with mutations in *ITGA2B* and *ITGB3* are indistinguishable in terms of phenotype or bleeding severity, as both varied appreciably, sometimes even among siblings.^8^ It has been observed that the majority of families have their own unique mutation; however, recurrence of some mutations indicate the presence of mutational hotspots.^9^ Missense variants in *ITGA2B* and *ITGB3* were studied in ∼32,000 alleles from 16,108 individuals and compared with 111 previously reported GT mutations. Respectively, 114 and 68 novel missense variants in *ITGA2B* and *ITGB3* were found, of which 96% were rare (minor allele frequency (MAF) < 0.1%).^12^ It is speculated that the availability of next-generation sequencing (NGS) will identify novel mutations in GT patients and carries. This will help in taking decision for the management of the disease, health care and treatment plan and to address the phenotype genotype inter- and intrafamilial variability.^9^ The heme panel is a custom-made panel by the Saudi Human Genome Program (SHGP). It includes a total of 393 genes, all of which have been implicated in blood and/or clotting disorders and listed as disease causing in OMIM.

We aimed in the current study to determine the genetic architecture of this disease in our patients and assess the feasibility and applicability of the SHGP hematology panel (heme panel) as a reliable diagnostic tool that will enable accurate, cost-effective and multiplex testing for patients with GT. We assembled a cohort of familial and sporadic cases diagnosed with GT and used this gene panel for new variant screening. The accessibility to both familial and sporadic cases has enabled us to conduct segregation analyses and population allele frequency calculations. We found previously reported mutations and new potential pathogenic variants in GT patients and family members.

## Results

### Gene coverage and target enrichment performance

The average number of variants detected/sampled was ~800 SNVs, and the overall coverage of targeted regions was about 98%. The target regions have an average depth of 248×. Supplementary Tables 1 and 2 summarize sequencing quality, alignment and overall coverage of target regions.

### Identification of mutations in causative genes

**Fig. 1:**
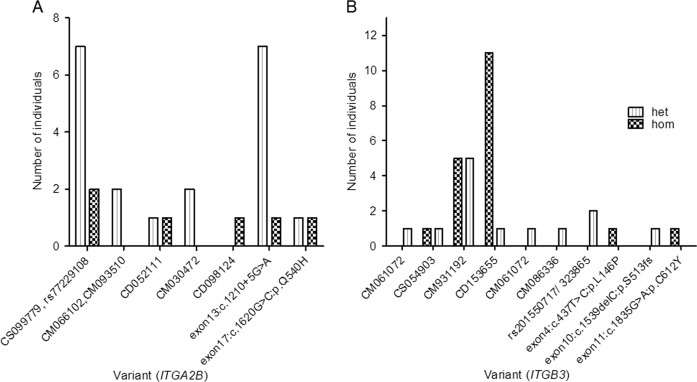
Variants count in our cohort. **a** *ITGA2B* and **b** *ITGB3*. Variants identified are presented on the *x-*axis. The *y*-axis depicts the number of individuals carrying the variant

Fifty-six individuals of our cohort harbored mutations in the known genes (Fig. 1). We identified 6 previously reported mutations in *ITGB3* (4 missense, 1 splice site and 1 frameshift) and 3 novel missense variants. In *ITGA2B*, we found 8 variants, 5 of these were previously reported mutations (3 missense, 1 splice site and 1 nonsense variants). Three of the identified variants in these two known genes were recurrent in our cases. In *ITGB3* the p. D243H was detected in individuals from 2 families (FAM-2 and FAM-3) and p. L705Cfs*4 was identified in individuals from families FAM-8 and FAM-13 and 6 sporadic cases. The *ITGA2B* splice variant c.1879-2A>G was recurrent in 3 families (FAM-5, FAM-9 and FAM-12). Two previously reported missense mutations were also recurrent in 4 sporadic cases. All the novel variants were predicted to be pathogenic using several tools. Homozygous mutations were identified in the majority of familial and sporadic cases, whereas heterozygous mutations were found in carriers and GT variant forms. There were some interfamilial differences with heterozygous diagnosed cases and a milder phenotype (Tables 1 and 2, and Supplementary Table 1).

**Table1:** Mutations Identified in *ITGA2B* and *ITGB3*

	Patient ID	Zygosity	Gene	Variant	HGMD or dbSNP
FAM-01^a^	GT-01	hom	*ITGA2B*	NM_000419:exon13:c.1210+5G>A	No HGMD
	GT-02	hom	*ITGA2B*	NM_000419:exon13:c.1210+5G>A	No HGMD
		hom	*ITGA2B*	NM_000419:exon13:c.1210+5G>A	No HGMD
	GT-03	hom	*ITGA2B*	NM_000419:exon13:c.1210+5G>A	No HGMD
	GT-04	hom	*ITGA2B*	NM_000419:exon13:c.1210+5G>A	No HGMD
	GT-05	hom	*ITGA2B*	NM_000419:exon13:c.1210+5G>A	No HGMD
	GT-07	hom	*ITGA2B*	NM_000419:exon13:c.1210+5G>A	No HGMD
	GT-08	het	*ITGA2B*	NM_000419:exon13:c.1210+5G>A	No HGMD
	GT-09	hom	*ITGA2B*	NM_000419:exon13:c.1210+5G>A	No HGMD
FAM-02^a^	GT-11	hom	*ITGB3*	NM_000212:exon5:c.727G>C:p.D243H	CM931192
	GT-12	hom	*ITGB3*	NM_000212:exon5:c.727G>C:p.D243H	CM931192
	GT-13^b^	het	*ITGB3*	NM_000212:exon5:c.727G>C:p.D243H	CM931192
	GT-14^b^	het	*ITGB3*	NM_000212:exon5:c.727G>C:p.D243H	CM931192
FAM-03^a^	GT-15	hom	*ITGB3*	NM_000212:exon5:c.727G>C:p.D243H	CM931192
	GT-16	hom	*ITGB3*	NM_000212:exon5:c.727G>C:p.D243H	CM931192
	GT-17	hom	*ITGB3*	NM_000212:exon5:c.727G>C:p.D243H	CM931192
	GT-18^b^	het	*ITGB3*	NM_000212:exon5:c.727G>C:p.D243H	CM931192
	GT-19^b^	het	*ITGB3*	NM_000212:exon5:c.727G>C:p.D243H	CM931192
	GT-20^b^	het	*ITGB3*	NM_000212:exon5:c.727G>C:p.D243H	CM931192
FAM-04	GT-21	hom	*ITGB3*	NM_000212:exon15:c.2302-1G>A	CS054903
	GT-22^c^	het	*ITGB3*	NM_000212:exon15:c.2302-1G>A	CS054903
FAM-05	GT-25	hom	*ITGA2B*	NM_000419:exon20:c.1879-2A>G	CS099779, rs77229108
	GT-26	hom	*ITGA2B*	NM_000419:exon20:c.1879-2A>G	CS099779, rs77229108
FAM-06	GT-27	hom	*ITGB3*	NM_000212:exon11:c.1835G>A:p.C612Y	No HGMD
	GT-28	NA	No DNA available for testing		
FAM-07	GT-29	hom	*ITGA2B*	NM_000419:exon17:c.1651C>T:p.R551W	CM066102, CM093510
	GT-30	hom	*ITGA2B*	NM_000419:exon17:c.1651C>T:p.R551W	CM066102, CM093510
FAM-08	GT-31	hom	*ITGB3*	NM_000212:exon13:c.2112delC:p.L705Cfs*4	CD153655
	GT-32	hom	*ITGB3*	NM_000212:exon13:c.2112delC:p.L705Cfs*4	CD153655
	GT-33	hom	*ITGB3*	NM_000212:exon13:c.2112delC:p.L705Cfs*4	CD153655
	GT-34	hom	*ITGB3*	NM_000212:exon13:c.2112delC:p.L705Cfs*4	CD153655
FAM-09	GT-35	hom	*ITGA2B*	NM_000419:exon20:c.1879-2A>G	CS099779, rs77229108
	GT-36	hom	*ITGA2B*	NM_000419:exon20:c.1879-2A>G	CS099779, rs77229108
	GT-37	hom	*ITGA2B*	NM_000419:exon20:c.1879-2A>G	CS099779, rs77229108
FAM-11	GT-43	hom	*ITGA2B*	NM_000419:exon17:c.1620G>C:p.Q540H	No HGMD
		hom	*ITGA2B*	NM_000419:exon17:c.1616T>G:p.L539R	CD052111
	GT-44^b^	het	*ITGA2B*	NM_000419:exon17:c.1620G>C:p.Q540H	No HGMD
		het	*ITGA2B*	NM_000419:exon17:c.1616T>G:p.L539R	CD052111
FAM-12^a^	GT-45^b^	het	*ITGA2B*	NM_000419:exon20:c.1879-2A>G	CS099779, rs77229108
	GT-46^b^	het	*ITGA2B*	NM_000419:exon20:c.1879-2A>G	CS099779, rs77229108
	GT-47	hom	*ITGA2B*	NM_000419:exon20:c.1879-2A>G	CS099779, rs77229108
	GT-48	hom	*ITGA2B*	NM_000419:exon20:c.1879-2A>G	CS099779, rs77229108
FAM-13	GT-49	hom	*ITGB3*	NM_000212:exon13:c.2112delC:p.L705Cfs*4	CD153655
	GT-50	hom	*ITGB3*	NM_000212:exon13:c.2112delC:p.L705Cfs*4	CD153655
	GT-51	hom	*ITGB3*	NM_000212:exon13:c.2112delC:p.L705Cfs*4	CD153655
	GT-52	hom	*ITGB3*	NM_000212:exon13:c.2112delC:p.L705Cfs*4	CD153655
Sporadic cases	GT-53	hom	*ITGB3*	NM_000212:exon13:c.2112delC:p.L705Cfs*4	CD153655
	GT-55	hom	*ITGB3*	NM_000212:exon13:c.2112delC:p.L705Cfs*4	CD153655
	GT-56	hom	*ITGA2B*	NM_000419:exon11:c.985G>T:p.V329F	CM030472
	GT-57	hom	*ITGB3*	NM_000212:exon13:c.2112delC:p.L705Cfs*4	CD153655
	GT-58	het	*ITGB3*	NM_000212:exon10:c.1539delC:p.S513fs	No HGMD
	GT-59^b^	het	*ITGB3*	NM_000212:exon13:c.2112delC:p.L705Cfs*4	CD153655
	GT-60^b^	het	*ITGB3*	NM_000212:exon13:c.2112delC:p.L705Cfs*4	CD153655
	GT-62	hom	*ITGA2B*	NM_000419:exon11:c.985G>T:p.V329F	CM030472
		het	*ITGB3*	NM_000212:exon10:c.1265G>A:p.S422N	CM061072
	GT-63	het	*ITGA2B*	NM_000419:exon4:c.558C>G:p.Y186X	CD098124
	GT-71^c^	het	*ITGA2B*	NM_000419:exon12:c.1142C>T:p.T381I	No HGMD
	GT-72^c^	het	*ITGB3*	NM_000212:exon5:c.662C>T:p.T221M	CM086336
	GT-73^c^	het	*ITGB3*	NM_000212:exon7:c.985A>G:p.N329D	rs201550717/ 323865
	GT-74^c^	het	*ITGB3*	NM_000212:exon7:c.985A>G:p.N329D	rs201550717/ 323865
	GT-76	hom	*ITGB3*	NM_000212:exon4:c.437T>C:p.L146P	No HGMD

*HGMD* Human Gene Mutation Database, *dbSNP* Single Nucleotide Polymorphism Database, *het* heterozygous, *hom* homozygous

^*^Family member, no definite diagnosis

^a^Consanguinity

^b^Unaffected family member (carrier)

^c^GT variant (heterozygous)

**Table 2:** Potential disease associated variants in genes with overlapping phenotype

Family ID	Patient ID	Zygosity	Gene	Variant	HGMD or dbSNP
FAM-11	GT-44^a^	het	*ITGA2*	NM_002203:exon9:c.958C>G:p.L320V	No HGMD
		het	*ITGA2*	NM_002203:exon9:c.967delA:p.K323fs	No HGMD
	GT-70^b^	het	*ITGA2*	NM_002203:exon14:c.1650A>C:p.E550D	No HGMD
	GT-75^b^	het	*ITGA2*	NM_002203:exon13:c.1535G>A:p.G512D	No HGMD
FAM-10^c^	GT-39^d^	het	*F8*	NM_000132:exon14:c.3780C>G:p.D1260E	CM960556
	GT-41^d^	het	*F8*	NM_000132:exon14:c.3780C>G:p.D1260E	CM960556
FAM-05	GT-24^d^	hom	*F8*	NM_000132:exon14:c.3780C>G:p.D1260E	CM960556
	GT-23^d^	hom	*VWF*	NM_000552:exon30:c.5191T>A:p.S1731T	CM012664

*HGMD* Human Gene Mutation Database, *dbSNP* Single Nucleotide Polymorphism Database, *het* heterozygous, *hom* homozygous

^a^Unaffected family member

^b^GT variant (heterozygous)

^c^Consanguinity

^d^Affected GT

### Potential disease-associated variants in other genes

The remaining cases with no previously reported mutation or a novel variant in the known genes were further analyzed. Therefore, using the same filtering steps, we proceeded with analysis to identify potential disease-related variants in other genes. In *ITGA2* (OMIM#192974), we found two heterozygous variants in two sporadic cases. We also found two mutations, described in the Human Gene Mutation Database (HGMD), in *F8* [NM_000132:exon14:c.3780C>G:p.D1260E (CM960556)] and *VWF* [NM_000552:exon30:c.5191T>A:p.S1731T (CM012664)], in individuals from two families. These four patients (GT-23, GT-24, GT-39 and GT-41) did not exhibit significant differences in their clinical presentations, compared to other cases in this study (Supplementary Table 3).

## Discussion

GT is characterized by a high degree of phenotypic heterogeneity, as evident by the wide variability in bleeding severity and αIIbβ3 expression. It is currently diagnosed and classified through clinical and biochemical parameters rather than through molecular testing. We assembled a cohort from familial cases with autosomal recessive mode of inheritance and sporadic cases. The samples exhibited phenotypic heterogeneity and variable severity of bleeding and were a mix of GT types and variants (Supplementary Table 3). Previously reported mutations and/or novel pathogenic variants in the known genes *ITGA3* and *ITGA2B* were identified in 56 out of 67 individuals including heterozygous carriers and GT-variants. We expanded the mutation spectrum in the remaining cases, negative for mutations in the two known genes, to include variants in *ITGA2*, where we identified potential disease-causing variants in two cases (Tables 1 and 2, and Supplementary Table 4). Validation by Sanger sequencing was carried out on a subset of samples, where DNA was available, as indicated in Tables 1 and 2.

Although extensive hematological and biochemical testing is performed for patients presenting clinically with symptoms and signs suggestive of GT, many do not reach a definitive diagnosis, due to the wide phenotypic and genotypic heterogeneity of the disease. The increasing availability of high-throughput DNA sequencing platforms provides efficient and accurate means for the understanding of variants leading to anomalies and malfunction of many proteins. Currently, NGS-based whole exome sequencing (WES) is successfully being used for molecular diagnosis of various Mendelian disorders.^13^ More gene panels have been designed to diagnose genetic disorders in general and inherited bleeding and coagulation disorders in particular.^14–16^ The ThromboGenomics consortium, for example, presents an affordable high-throughput molecular diagnostic tool of a panel comprised of 76 genes implicated in inherited bleeding, thrombotic and platelet disorders.^17^

The SHGP heme panel is believed to be suitable and extensive (covering 393 genes) to identify variants in GT-associated genes and can be expanded to include other bleeding/hematological disorders. The availability of such validated gene panels facilitates accurate, cost-effective and fast diagnosis and consequently better management.^18,19^ Targeted gene panels surpass exome sequencing by overcoming some of its limitations. They generate fewer variants compared to WES, therefore making the variant selection, validation and interpretation more efficient and easier to handle. More importantly, since the analysis is limited to genes related to selected hematological conditions, incidental findings are reduced. The SHGP heme panel also allows multiplexing of 33 samples testing pipeline with an incorporated locally defined normal control. The panel’s design covers exon intron boundaries as no deep intronic variants are commonly known to be associated with such disorders.^20^ Finally, gene panels coverage can be as high as 100% when coupled with Sanger sequencing.^21^ In the case of GT, where mutations occur only in two known genes, the heme panel presents itself as an attractive and accurate tool for clinical diagnosis. Given the high heterogeneity in clinical presentation, the panel would provide extensive and thorough analysis of variants present in the known causative genes and it is also useful for identifying SNVs in other genes implicated in hematological or overlapping bleeding disorders. A recent study, using targeted NGS to find genetic variants in GT patients, identified mutations in other genes in addition to *ITGA2B* and *ITGB3*, such as *RUNX1*, *HPS4*, *MYH9*, *ACTN1*, *HPS3* and *SETBP1*.^22^

For patients with clear biochemical and laboratory markers indicative of GT diagnosis, it may be unnecessary to run samples for WES. A targeted panel is a sufficiently reliable confirmational diagnostic test,^18,19^ especially in cases that present with mild to moderate bleeding severity and where biochemical markers are not conclusive. It can also be used for carrier testing for family members of affected individuals to allow better management and genetic counseling.

Unlimited to GT, but to all inherited platelet disorders, establishing a conclusive molecular diagnosis should be encouraged in order to provide the best patient care in terms of management, prognosis and counseling. In the majority of inherited platelet disorders, a single candidate gene is not clearly apparent from standard laboratory tests. Consequently, a molecular diagnosis of inherited platelet disorders is offered in only a minority of patients, since a single gene cannot be easily nominated to be a candidate disease causing one.^20^ Hence, genetic testing is primarily used to confirm uncertain clinical diagnosis, usually attributed to a limited number of genes.

Consequences of variants in *ITGA2B* and *ITGB3* in GT patients were reported to cause lack of function, prevention of αIIbβ3 expression, and are associated with activated integrin defects and macrothrombocytopenia. There are also amino acid substitutions that give rise to human platelet alloantigen systems without affecting αIIbβ3 expression or function. On the other hand, many GT patients harboring heterozygous mutations and presenting with intermediate levels of αIIbβ3 are asymptomatic and show a full platelet aggregation response.^23^

In our GT cohort, some families harbored a unique variant while other families/individuals shared recurrent variants, making these potential gene “hotspot” as reported in other studies.^4^ It is noteworthy that the variants identified in this study have a MAF < 1% in the local normal control data set comprising 3206 exomes. The variants leading to missense, nonsense and frameshift changes are present on different protein domains of ITGA2B and ITGB3 (Supplementary Figure 2). The type and position of the mutation was shown to influence the residual functional response.^11^ The stimulated platelets in GT-variants are incapable to bind soluble Fg or antibodies responsible for recognizing activation-dependent determinants on αIIbβ3.^4^

We expanded our search for pathogenic variants in samples where no *ITGB3* or *ITGA2B* disease-related mutations were found. We were able to identify potential pathogenic variants in *ITGA2*, *VWF* and *F8* genes. In *ITGA2*, we identified three missense variants and one frameshift. Of the novel *ITGA2* variants, all were possibly pathogenic as predicted by Combined Annotation–Dependent Depletion (CADD) and other pathogenicity prediction tools. The two missense variants p.G512D and p.E550D have a CADD score of 32 (Table 2 and Supplementary Table 2). There are reports in the literature linking the Integrin Subunit Alpha 2, *ITGA2*, with prolonged bleeding, thrombocytopenia and defective adhesion to collagen and sub-endothelium.^24–26^ In other reports, the association of variants in *ITGA2* with increased thrombotic risk showed contradicting findings.^27^ Functional assessment of all the newly identified potential disease variants is required. The reported detection yield of gene panels for inherited diseases varies widely (24–95%),^19,28–30^ supporting the idea that gene panels such as the one we used delivers an accurate and affordable sequencing of inherited disease-causing genes.^31^

In our cohort, we were able to identify mutations and potentially pathogenic variants in ~92.5% of our cases (62 out of 67 patients and carrier family members, 56 of those harbored mutations in the 2 reported genes). In this calculation we excluded wild type unaffected family members from the total NGS cases. We found a total of 17 variants in *ITGB3* and *ITGA2B* including 6 that were not previously reported in GT and we expanded the genotypic association with GT to include variants in *ITGA2*, where we found new variants, which predicted to be pathogenic by multiple tools. Targeted NGS panel testing was found to be useful and cost effective for definite diagnosis and carrier testing, leading to better healthcare management and counseling in different inherited disorders.^18^ Further studies on larger number of samples using this technology will expand the mutation spectrum in GT.

## Methods

### Patients and families

The study was approved by the research advisory council in King Faisal Specialist Hospital and Research Centre (KFSHRC), Research Advisory Council (RAC) approval number 2130036. A total of 72 individuals from 14 familial and sporadic cases along with their family members (whenever possible) with an apparent autosomal recessive inheritance and previous history of GT were evaluated and enrolled. Families are from different regions in Saudi Arabia, where there is a considerable degree of consanguinity and endogamy. All clinical and laboratory data were collected from medical records (Supplementary Table 1). Written informed consent was obtained from all human participants (patients and family members).

### DNA extraction and Sanger sequencing

DNA was extracted from peripheral blood samples from patients and other family members (when available), using the Gentra Puregene Blood Extraction kit (Qiagen, Valencia, California). As for validation by Sanger sequencing, PCR and sequencing was performed as described previously.^18,32^

### Next-generation sequencing and analysis

Samples were processed for targeted sequencing using the available SHGP heme panel of 393 genes. Briefly, 10 ng of DNA were amplified using two Primer Pools (SHGP heme panel), AmpliSeqHiFi mix (Thermo Fisher, Carlsbad, CA, USA) and 16 amplification cycles. The resulting PCR pools for each sample were combined for digestion with FuPa reagent (Thermo Fisher, Carlsbad, CA, USA). The pooled amplicons were then ligated to individual universal adaptors and purified. After purification, libraries were quantitated by quantitative PCR, normalized to 100 pM and pooled (33 samples/reaction) for emulsion PCR using the Ion OneTouch System. This was followed by enrichment using the Ion OneTouch ES according to the manufacturer’s instructions (Thermo Fisher, Carlsbad, CA, USA). The template-positive Ion PITM Ion Sphere particles were then sequenced on the Ion Proton instrument (Thermo Fisher, Carlsbad, CA, USA) in the SHGP Lab at the Department of Genetics in KFSHRC.

### Primary pipeline, filtering and analysis

The quality of each read was checked and confirmed, and low reads (<20) were excluded as a primary quality check. The reads were then aligned to the hg19 human genome reference sequence using the Ion Torrent Suite program (Thermo Fisher; https://github.com/iontorrent/TS). This was followed by variant calling through the Torrent Suite Variant Caller TVC program. All variants were annotated using a combination of public and in-house databases. The public databases were obtained from the ANNOVAR package in addition to other available datasets, such as the Human Gene Mutation Database (HGMD).^33^ The analysis pipeline was designed to filter out all non-relevant variants based on quality, functional characteristics and frequency in the databases including the local SHGP database. Further analysis focused on the identification of all nonsense, frameshift and canonical splice site variants in genes related to GT and overlapping bleeding disorders, including previously reported variants with an HGMD ID.^18,32^ Multiple pathogenicity prediction tools were used as described below. All identified variants were validated by conventional Sanger sequencing and segregation analysis was performed when possible in a subset of samples.

### Variant prioritization and mutation detection

Multiple web-based bioinformatics tools were used to classify the predicted pathogenicity of the variants identified (Supplementary Table 2). First, we have used the web-based tool CADD, which is used for integrating diverse genome annotations in order to score SNV or small insertion–deletion (indel) events. Deleterious variants are correlated with both molecular functionality and pathogenicity.^34^ We then expanded our analysis through a consensus classifier combining five best performing prediction methods, PredictSNP2. These computational tools are complimented by experimental annotations from eight databases. PredictSNP2 yields its output in the form of a normalized confidence score presented as a percentage.^35^ Finally, we added rank scores calculated by Variant Ranker, a web-tool that ranks both coding and noncoding variants. It is based on a combination of factors that takes into consideration novelty and uniqueness of the variant type, and its effect and annotation information. The tool is designed to implement and aggregate several prediction algorithms and conservation scores, allelic frequencies, clinical information and additional annotations using accessible databases via ANNOVAR.^36^ We used the term “variant” to describe novel SNVs identified in this study, and the term “mutation” to refer to previously reported SNVs with HGMD/ClinVar entry.

### Reporting summary

Further information on experimental design is available in the Nature Research Reporting Summary linked to this article.

## Supplementary information

Supplemental Material

Reporting Summary

## Data Availability

All data supporting the results reported in this article can be found in the Supplementary Material.

## Supplementary information

**Supplementary information** accompanies the paper on the *npj Genomic Medicine* website (10.1038/s41525-019-0079-6).

## References

[1] George JN, Caen JP, Nurden AT (1990). Glanzmann’s thrombasthenia: the spectrum of clinical disease. Blood.

[2] Nurden AT (1999). Inherited abnormalities of platelets. Thromb. Haemost..

[3] Bellucci S, Caen J (2002). Molecular basis of Glanzmann’s thrombasthenia and current strategies in treatment. Blood Rev..

[4] Nurden AT, Pillois X, Wilcox DA (2013). Glanzmann thrombasthenia: state of the art and future directions. Semin. Thromb. Hemost..

[5] Al-Fawaz IM (1996). Hereditary bleeding disorders in Riyadh, Saudi Arabia. Ann. Saudi Med..

[6] Nounou R, Spence D (1993). Glanzmann’s thrombasthenia with mild von Willebrand’s disease. J. Clin. Pathol..

[7] Ai-Barghouthi SK, Ai-Othman A, Lardhi A (1997). Glanzmann’s thrombasthenia-spectrum of clinical presentation on Saudi patients in the Eastern Province. J. Fam. Community Med..

[8] Nurden AT (2015). Expanding the mutation spectrum affecting alphaIIbbeta3 integrin in Glanzmann thrombasthenia: screening of the ITGA2B and ITGB3 genes in a large international cohort. Hum. Mutat..

[9] Nurden AT, Pillois X, Nurden P (2012). Understanding the genetic basis of Glanzmann thrombasthenia: implications for treatment. Expert Rev. Hematol..

[10] Wilcox, D. A. Glanzmann Thrombasthenia Database, http://sinaicentral.mssm.edu/intranet/research/glanzmann (2018).

[11] Nurden AT, Fiore M, Nurden P, Pillois X (2011). Glanzmann thrombasthenia: a review of ITGA2B and ITGB3 defects with emphasis on variants, phenotypic variability, and mouse models. Blood.

[12] Buitrago L (2015). alphaIIbbeta3 variants defined by next-generation sequencing: predicting variants likely to cause Glanzmann thrombasthenia. Proc. Natl. Acad. Sci. USA.

[13] Tucker T, Marra M, Friedman JM (2009). Massively parallel sequencing: the next big thing in genetic medicine. Am. J. Hum. Genet..

[14] Monies D (2017). The landscape of genetic diseases in Saudi Arabia based on the first 1000 diagnostic panels and exomes. Hum. Genet..

[15] Bastida JM (2016). Design and application of a 23-gene panel by next-generation sequencing for inherited coagulation bleeding disorders. Haemophilia.

[16] Tekin D (2016). A next-generation sequencing gene panel (MiamiOtoGenes) for comprehensive analysis of deafness genes. Hear. Res..

[17] Simeoni I (2016). A high-throughput sequencing test for diagnosing inherited bleeding, thrombotic, and platelet disorders. Blood.

[18] Mustafa, A. E. et al. Validation of ion torrent(TM) inherited disease panel with the PGM(TM) sequencing platform for rapid and comprehensive mutation detection. *Genes* **9**, 267, 10.3390/genes9050267 (2018). 10.3390/genes9050267 PMC5977207 29789446

[19] Saudi Mendeliome G (2015). Comprehensive gene panels provide advantages over clinical exome sequencing for Mendelian diseases. Genome Biol..

[20] Lentaigne C (2016). Inherited platelet disorders: toward DNA-based diagnosis. Blood.

[21] Sun Y (2015). Next-generation diagnostics: gene panel, exome, or whole genome?. Hum. Mutat..

[22] Miao LZ (2018). [Molecular analysis of gene mutations in eight patients with Glanzmann’s thrombasthenia]. Zhonghua Yi Xue Za Zhi.

[23] Nurden AT (2017). Should studies on Glanzmann thrombasthenia not be telling us more about cardiovascular disease and other major illnesses?. Blood Rev..

[24] Nieuwenhuis HK, Akkerman JW, Houdijk WP, Sixma JJ (1985). Human blood platelets showing no response to collagen fail to express surface glycoprotein Ia. Nature.

[25] Nieuwenhuis HK, Sakariassen KS, Houdijk WP, Nievelstein PF, Sixma JJ (1986). Deficiency of platelet membrane glycoprotein Ia associated with a decreased platelet adhesion to subendothelium: a defect in platelet spreading. Blood.

[26] Noris P (2006). Autosomal dominant thrombocytopenias with reduced expression of glycoprotein Ia. Thromb. Haemost..

[27] Martinez C (2009). Genotype-phenotype relationship for six common polymorphisms in genes affecting platelet function from 286 healthy subjects and 160 patients with mucocutaneous bleeding of unknown cause. Br. J. Haematol..

[28] Mori T (2017). Comprehensive genetic testing approach for major inherited kidney diseases, using next-generation sequencing with a custom panel. Clin. Exp. Nephrol..

[29] Carrigan M (2016). Panel-based population next-generation sequencing for inherited retinal degenerations. Sci. Rep..

[30] Al-Mousa H (2016). Unbiased targeted next-generation sequencing molecular approach for primary immunodeficiency diseases. J. Allergy Clin. Immunol..

[31] Pua CJ (2016). Development of a comprehensive sequencing assay for inherited cardiac condition genes. J. Cardiovasc Transl. Res.

[32] Al-Mubarak B (2017). Whole exome sequencing reveals inherited and de novo variants in autism spectrum disorder: a trio study from Saudi families. Sci. Rep..

[33] Stenson PD (2017). The Human Gene Mutation Database: towards a comprehensive repository of inherited mutation data for medical research, genetic diagnosis and next-generation sequencing studies. Hum. Genet..

[34] Kircher M (2014). A general framework for estimating the relative pathogenicity of human genetic variants. Nat. Genet..

[35] Bendl J (2016). PredictSNP2: a unified platform for accurately evaluating SNP effects by exploiting the different characteristics of variants in distinct genomic regions. PLoS Comput. Biol..

[36] Alexander J, Mantzaris D, Georgitsi M, Drineas P, Paschou P (2017). Variant Ranker: a web-tool to rank genomic data according to functional significance. BMC Bioinforma..

