# Supplementary material for: Molecular yield of targeted sequencing for Glanzmann thrombasthenia patients
Source: NPJ Genom Med. 2019 Feb 14;4:4. doi: 10.1038/s41525-019-0079-6 (PMC6375963; doi:10.1038/s41525-019-0079-6)
Supplement: Supplementary file 1 — Supplemental Material [file 41525_2019_79_MOESM1_ESM.docx]

**
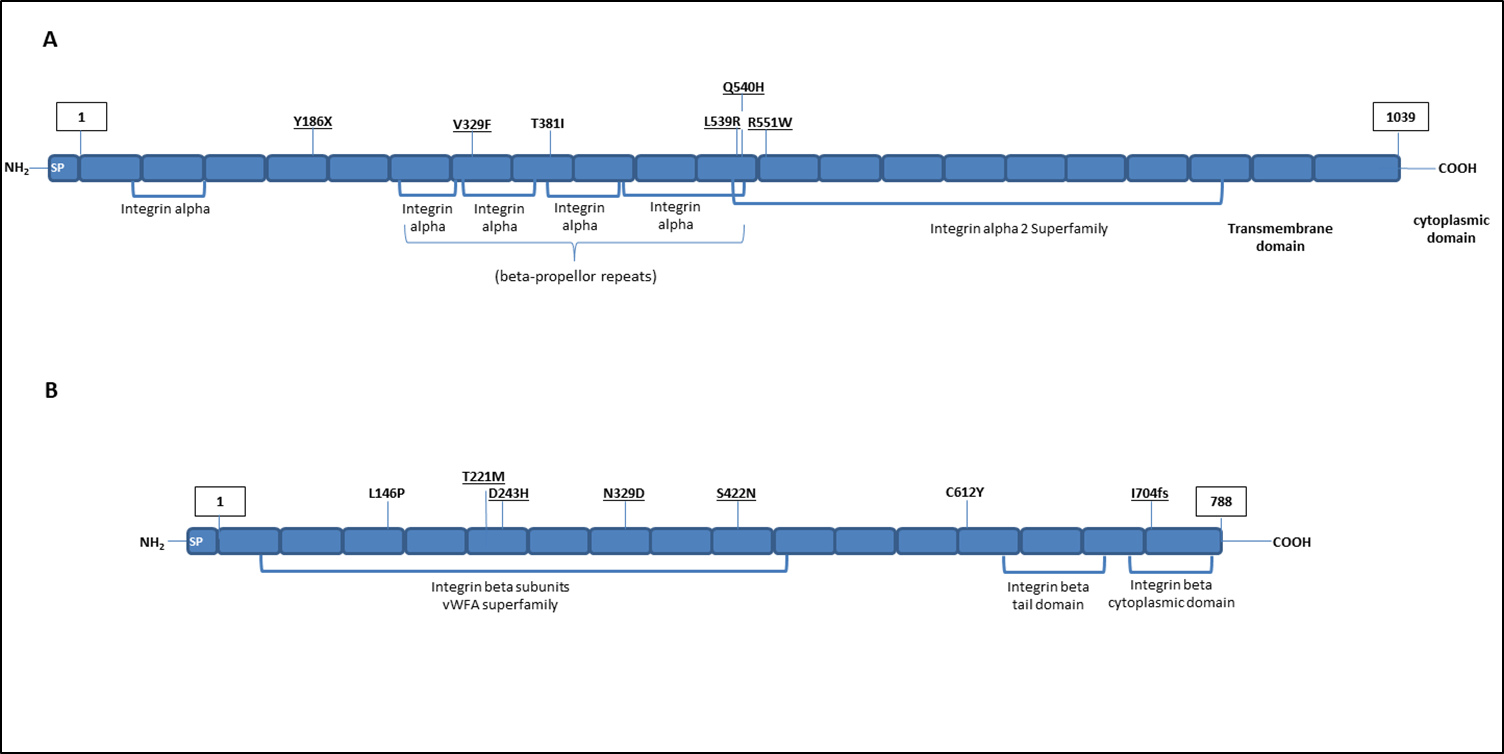
**

**Supplementary Figure 1**: Variants presentation across the protein domains of ITGA2B and ITGB3. **A)** ITGA2B , **B)** ITGB3. Underlined variants indicate known mutations in HGMD. Figure adapted from^1-5^.

**Supplementary Table 1: Sequencing depth, coverage and other metrics for exomes in a subset of samples**

| Sample ID | Number of Bases at Q0 | Number of Bases at Q20 | Mean Read Length at Q0 | Mean Read Length at Q20 | Reads at Q0 | Reads at Q20 | Number of mapped reads | Percent reads on target | Number of amplicons | Average depth | Target base coverage at 1x | Target base coverage at 20x |
| --- | --- | --- | --- | --- | --- | --- | --- | --- | --- | --- | --- | --- |
| GT-01 | 300580793 | 194314099 | 170 | 109 | 1767656 | 1767656 | 1752053 | 96.68% | 7472 | 192 | 98.34% | 93.22% |
| GT-03 | 245774054 | 167595913 | 172 | 117 | 1425268 | 1425268 | 1408972 | 96.36% | 7472 | 156.8 | 98.13% | 91.97% |
| GT-04 | 303877072 | 206327854 | 170 | 115 | 1781196 | 1781196 | 1759117 | 96.13% | 7472 | 193.4 | 98.43% | 93.63% |
| GT-05 | 523308948 | 347384805 | 167 | 111 | 3124444 | 3124444 | 3089201 | 96.67% | 7472 | 335.3 | 98.37% | 94.15% |
| GT-07 | 617505879 | 386467231 | 162 | 101 | 3803844 | 3803844 | 3738143 | 94.53% | 7472 | 387.2 | 98.56% | 94.16% |
| GT-08 | 261640158 | 173645357 | 169 | 112 | 1543143 | 1543143 | 1524986 | 95.92% | 7472 | 166.4 | 98.26% | 93.14% |
| GT-11 | 540958453 | 355937801 | 167 | 110 | 3225574 | 3225574 | 3189249 | 96.23% | 7472 | 345.3 | 98.42% | 94.02% |
| GT-12 | 674082259 | 448611563 | 167 | 111 | 4025942 | 4025942 | 3982771 | 96.87% | 7472 | 432.9 | 98.39% | 94.61% |
| GT-13 | 337140220 | 232727233 | 168 | 116 | 2005895 | 2005895 | 1984134 | 97.07% | 7472 | 216.2 | 96.91% | 68.52% |
| GT-14 | 282938956 | 185406760 | 167 | 109 | 1691834 | 1691834 | 1675625 | 96.94% | 7472 | 181 | 97.44% | 90.32% |
| GT-15 | 285207277 | 186134523 | 168 | 110 | 1690367 | 1690367 | 1672615 | 96.99% | 7472 | 182.4 | 97.75% | 90.98% |
| GT-16 | 301293235 | 197970907 | 169 | 111 | 1779140 | 1779140 | 1763699 | 97.31% | 7472 | 193.3 | 97.77% | 91.27% |
| GT-17 | 921640190 | 596014318 | 167 | 108 | 5503543 | 5503543 | 5448081 | 97.06% | 7472 | 591 | 98.50% | 94.85% |
| GT-18 | 283326223 | 179730186 | 168 | 106 | 1683697 | 1683697 | 1667630 | 96.98% | 7472 | 181.2 | 97.82% | 90.53% |
| GT-19 | 314630443 | 207738061 | 167 | 110 | 1872847 | 1872847 | 1853553 | 96.96% | 7472 | 201.3 | 97.84% | 92.05% |
| GT-20 | 405603370 | 267372320 | 169 | 111 | 2388088 | 2388088 | 2366695 | 97.06% | 7472 | 259.8 | 98.15% | 92.94% |
| GT-21 | 306191172 | 198458485 | 166 | 108 | 1837201 | 1837201 | 1819350 | 96.11% | 7472 | 194.8 | 97.80% | 91.33% |
| GT-22 | 349700722 | 216898301 | 165 | 102 | 2119358 | 2119358 | 2099060 | 95.94% | 7472 | 222.1 | 98.16% | 91.94% |
| GT-23 | 371482071 | 237966268 | 167 | 107 | 2218952 | 2218952 | 2199750 | 96.83% | 7472 | 237.6 | 98.13% | 92.30% |
| GT-24 | 356092908 | 235844996 | 169 | 112 | 2104618 | 2104618 | 2084977 | 96.60% | 7472 | 227.5 | 97.91% | 91.92% |
| GT-25 | 404509177 | 259878503 | 166 | 107 | 2427284 | 2427284 | 2403444 | 96.01% | 7472 | 257.1 | 98.16% | 92.96% |
| GT-26 | 899433868 | 583979145 | 165 | 107 | 5442913 | 5442913 | 5360307 | 95.40% | 7472 | 568.8 | 98.70% | 95.35% |
| GT-27 | 368334457 | 236149552 | 167 | 107 | 2196691 | 2196691 | 2179522 | 96.67% | 7472 | 235.3 | 97.82% | 91.98% |
| GT-29 | 248870521 | 161512223 | 167 | 108 | 1485411 | 1485411 | 1467333 | 94.87% | 7472 | 156.6 | 98.32% | 92.86% |
| GT-30 | 655152958 | 426288667 | 163 | 106 | 4009156 | 4009156 | 3941751 | 94.57% | 7472 | 412 | 98.85% | 95.05% |
| GT-31 | 347456155 | 226717743 | 168 | 109 | 2067337 | 2067337 | 2044664 | 96.14% | 7472 | 221.2 | 97.86% | 91.64% |
| GT-32 | 300477422 | 196160911 | 167 | 109 | 1789106 | 1789106 | 1772330 | 96.89% | 7472 | 192 | 97.73% | 92.08% |
| GT-33 | 319989386 | 204598200 | 166 | 106 | 1926085 | 1926085 | 1906982 | 96.13% | 7472 | 203.5 | 97.92% | 92.23% |
| GT-34 | 362242916 | 291150998 | 172 | 138 | 2104617 | 2104617 | 2094802 | 93.31% | 7472 | 222.5 | 99.01% | 93.46% |
| GT-35 | 311060928 | 206265685 | 170 | 112 | 1827197 | 1827197 | 1812180 | 97.45% | 7472 | 200 | 97.53% | 91.30% |
| GT-36 | 357963731 | 242477637 | 170 | 115 | 2103641 | 2103641 | 2086294 | 97.30% | 7472 | 229.8 | 97.77% | 91.57% |
| GT-37 | 251085722 | 169049496 | 168 | 113 | 1487710 | 1487710 | 1474569 | 96.94% | 7472 | 160.8 | 97.71% | 91.85% |
| GT-38 | 484503849 | 313725027 | 166 | 107 | 2913399 | 2913399 | 2877001 | 95.52% | 7472 | 306.1 | 98.47% | 93.15% |
| GT-39 | 275839367 | 186175179 | 169 | 114 | 1630175 | 1630175 | 1613784 | 96.48% | 7472 | 176.1 | 98.18% | 90.59% |
| GT-40 | 543554683 | 353148979 | 166 | 108 | 3267039 | 3267039 | 3230618 | 96.21% | 7472 | 346.2 | 98.56% | 94.48% |
| GT-41 | 315287895 | 214122833 | 171 | 116 | 1840274 | 1840274 | 1823288 | 96.90% | 7472 | 201.9 | 98.42% | 92.80% |
| GT-42 | 302021819 | 197653785 | 168 | 110 | 1792396 | 1792396 | 1768111 | 96.46% | 7472 | 192.8 | 98.22% | 93.02% |
| GT-43 | 570989776 | 370589807 | 164 | 107 | 3462699 | 3462699 | 3414982 | 95.44% | 7472 | 360.9 | 98.55% | 93.93% |
| GT-44 | 310491755 | 206182916 | 168 | 111 | 1842785 | 1842785 | 1818034 | 94.89% | 7472 | 196.1 | 98.24% | 93.44% |
| GT-45 | 279557295 | 191568655 | 171 | 117 | 1631179 | 1631179 | 1612454 | 94.65% | 7472 | 175.5 | 98.22% | 92.07% |
| GT-46 | 409646995 | 280928527 | 173 | 118 | 2367106 | 2367106 | 2346168 | 96.89% | 7472 | 262.5 | 98.51% | 94.10% |
| GT-47 | 259136785 | 169614674 | 168 | 110 | 1538213 | 1538213 | 1522839 | 96.23% | 7472 | 165.2 | 98.04% | 90.82% |
| GT-48 | 485376088 | 325058497 | 167 | 112 | 2891873 | 2891873 | 2862259 | 96.35% | 7472 | 309.7 | 98.39% | 94.25% |
| GT-49 | 548042758 | 429297469 | 172 | 135 | 3171467 | 3171467 | 3155713 | 93.57% | 7472 | 336.7 | 99.31% | 96.64% |
| GT-50 | 149158204 | 117096600 | 171 | 134 | 870183 | 870183 | 865465 | 90.04% | 7472 | 88.65 | 98.40% | 80.86% |
| GT-51 | 115398214 | 89572957 | 173 | 134 | 667006 | 667006 | 664444 | 95.53% | 7472 | 71.81 | 98.41% | 86.33% |
| GT-52 | 325572554 | 209256424 | 164 | 105 | 1979005 | 1979005 | 1955455 | 96.43% | 7472 | 208 | 97.14% | 89.20% |
| GT-53 | 566796454 | 475929879 | 188 | 158 | 3003830 | 3003830 | 2989945 | 96.22% | 7472 | 358.6 | 99.18% | 96.83% |
| GT-54 | 10186267 | 8502137 | 183 | 152 | 55653 | 55653 | 55222 | 92.48% | 7472 | 6.204 | 84.18% | 6.00% |
| GT-55 | 342389568 | 220972572 | 166 | 107 | 2054040 | 2054040 | 2031951 | 96.16% | 7472 | 217.8 | 98.12% | 92.06% |
| GT-56 | 382616358 | 250060760 | 167 | 109 | 2291042 | 2291042 | 2270687 | 96.36% | 7472 | 244.1 | 98.14% | 93.16% |
| GT-57 | 704700871 | 545257649 | 172 | 133 | 4096724 | 4096724 | 4078109 | 95.25% | 7472 | 437.9 | 99.46% | 97.19% |
| GT-58 | 313880078 | 165303060 | 144 | 76 | 2172779 | 2172779 | 2135758 | 95.33% | 7472 | 197.1 | 97.31% | 86.82% |
| GT-59 | 314086117 | 192004322 | 166 | 101 | 1883056 | 1883056 | 1867867 | 96.57% | 7472 | 200.4 | 97.74% | 90.19% |
| GT-60 | 272702006 | 182405720 | 171 | 114 | 1590964 | 1590964 | 1574321 | 95.63% | 7472 | 173.1 | 98.11% | 91.86% |
| GT-61 | 384972672 | 253135878 | 167 | 110 | 2298716 | 2298716 | 2274590 | 96.04% | 7472 | 245.1 | 98.17% | 92.58% |
| GT-62 | 244916239 | 155065116 | 165 | 104 | 1482291 | 1482291 | 1466804 | 96.62% | 7472 | 156.3 | 96.95% | 88.76% |
| GT-63 | 335858205 | 219607101 | 168 | 109 | 1997869 | 1997869 | 1978726 | 96.14% | 7472 | 213.6 | 97.85% | 91.64% |
| GT-64 | 1041032974 | 792127799 | 171 | 130 | 6063814 | 6063814 | 6036696 | 95.93% | 7472 | 650.9 | 99.31% | 97.68% |
| GT-65 | 451925172 | 375668001 | 184 | 153 | 2453523 | 2453523 | 2433360 | 93.32% | 7472 | 277.2 | 97.21% | 84.92% |

**Supplementary Table 2: The number of single point variants and insertion or deletion of bases identified in a subset of samples**

| **Sample ID** | **Variant Count** | **SNP** | **INDEL** |
| --- | --- | --- | --- |
| HDS/GT-26.hds.csv | 1712 | 1613 | 99 |
| HDS/GT-24.hds.csv | 1463 | 1369 | 94 |
| HDS/GT-23-1.hds.csv | 1376 | 1285 | 91 |
| HDS/GT-25.hds.csv | 1363 | 1275 | 88 |
| HDS/GT-27.hds.csv | 1477 | 1381 | 96 |
| HDS/GT-21.hds.csv | 1406 | 1309 | 97 |
| HDS/GT-22.hds.csv | 1557 | 1464 | 93 |
| HDS/GT-61.hds.csv | 1474 | 1389 | 85 |
| HDS/GT-56.hds.csv | 1373 | 1288 | 85 |
| HDS/GT-63.hds.csv | 1360 | 1290 | 70 |
| HDS/GT-59.hds.csv | 1400 | 1299 | 101 |
| HDS/GT-55.hds.csv | 1340 | 1252 | 88 |
| HDS/GT-31.hds.csv | 1372 | 1300 | 72 |
| HDS/GT-33.hds.csv | 1426 | 1332 | 94 |
| HDS/ GT-32.hds.csv | 1363 | 1282 | 81 |
| HDS/GT-37.hds.csv | 1296 | 1223 | 73 |
| HDS/GT-36.hds.csv | 1338 | 1243 | 95 |
| HDS/GT-35.hds.csv | 1389 | 1310 | 79 |
| HDS/GT-16.hds.csv | 1367 | 1276 | 91 |
| HDS/GT-17.hds.csv | 1424 | 1331 | 93 |
| HDS/GT-19.hds.csv | 1434 | 1351 | 83 |
| HDS/GT-15.hds.csv | 1362 | 1283 | 79 |
| HDS/GT-20.hds.csv | 1378 | 1289 | 89 |
| HDS/GT-18.hds.csv | 1369 | 1297 | 72 |
| HDS/GT-40.hds.csv | 1488 | 1373 | 115 |
| HDS/GT-48.hds.csv | 1409 | 1310 | 99 |
| HDS/GT-05.hds.csv | 1461 | 1364 | 97 |
| HDS/GT-12.hds.csv | 1444 | 1362 | 82 |
| HDS/GT-11.hds.csv | 1426 | 1348 | 78 |
| HDS/GT-14.hds.csv | 1358 | 1267 | 91 |
| HDS/GT-13.hds.csv | 1275 | 1222 | 53 |
| HDS/GT-43.hds.csv | 1559 | 1461 | 98 |
| HDS/GT-62.hds.csv | 1309 | 1234 | 75 |
| HDS/GT-30.hds.csv | 1561 | 1461 | 100 |
| HDS/GT-52.hds.csv | 1318 | 1240 | 78 |
| HDS/GT-58.hds.csv | 1333 | 1233 | 100 |
| HDS/GT-38.hds.csv | 1648 | 1558 | 90 |
| HDS/GT-39.hds.csv | 1311 | 1235 | 76 |
| HDS/GT-41.hds.csv | 1400 | 1313 | 87 |
| HDS/GT-42.hds.csv | 1400 | 1315 | 85 |
| HDS/GT-47.hds.csv | 1328 | 1232 | 96 |
| HDS/GT-45.hds.csv | 1627 | 1523 | 104 |
| HDS/GT-46.hds.csv | 1367 | 1277 | 90 |
| HDS/GT-03.hds.csv | 1403 | 1322 | 81 |
| HDS/GT-01.hds.csv | 1539 | 1441 | 98 |
| HDS/GT-04.hds.csv | 1495 | 1418 | 77 |
| HDS/GT-07.hds.csv | 1629 | 1555 | 74 |
| HDS/GT-08.hds.csv | 1483 | 1395 | 88 |
| HDS/GT-44.hds.csv | 1536 | 1451 | 85 |
| HDS/GT-29.hds.csv | 1340 | 1265 | 75 |
| HDS/GT-60.hds.csv | 1377 | 1311 | 66 |
| HDS/GT-53.hds.csv | 1256 | 1180 | 76 |
| HDS/GT-65.hds.csv | 1105 | 1077 | 28 |
| HDS/GT-54.hds.csv | 478 | 475 | 3 |
| HDS/GT-34.hds.csv | 1301 | 1247 | 54 |
| HDS/GT-49.hds.csv | 1345 | 1276 | 69 |
| HDS/GT-64.hds.csv | 1401 | 1340 | 61 |
| HDS/GT-50.hds.csv | 1299 | 1252 | 47 |
| HDS/GT-51.hds.csv | 1256 | 1204 | 52 |
| HDS/GT-57.hds.csv | 1304 | 1248 | 56 |

SNP: single nucleotide polymorphism

INDEL: insertion or deletion of bases

**Supplementary Table 3: Summary of selected biochemical parameters**

| Patient ID | Gender | GT Type^*^ | Bleeding  severity^#^ | Hgb | Nasal/Gum# | PLT (X109) | MCV | MPV | PFA^$^ 100 | APTT | collagen | Flow Cytometry^^^ | |
| --- | --- | --- | --- | --- | --- | --- | --- | --- | --- | --- | --- | --- | --- |
|  |  |  |  |  |  |  |  |  |  |  |  | CD41 | CD61 |
| GT-01 | M | 1 | 3 | 113 | 1-2/1-2 | 353 | 79.4 | 8 | >235 | 34.2 | 2 | 1 | 1 |
| GT-02 | M | NA | NA | NA | 1-2/1-2 | NA | NA | NA | NA | NA | NA | 1 | 1 |
| GT-03 | M | 1 | NA | 108 | 1-2/1-2 | 294 | 72.4 | 10.4 | >300 | 31.4 | 1 | 1 | 1 |
| GT-04 | M | 1 | 1 | 81 | 1-2/1-2 | 347 | 61.4 | 7.7 | >233 | 34.5 | 2 | 1 | 1 |
| GT-05 | M | 1 | 1 | 114 | 1-2/1-2 | 255 | 77.7 | 8.3 | 240 | 29.2 | 2 | 1 | 1 |
| GT-06^$^ | F | 1 | 1 | 106 | 1-2/1-2 | 305 | 69.3 | 9.5 | 263 | 39.9 | 2 | 1 | 1 |
| GT-07 | M | 1 | 1 | 109 | 1-2/1-2 | 338 | 83.5 | 9.5 | >234 | 27.8 | 2 | 1 | 1 |
| GT-08 | F | NA | NA | NA | 1-2/1-2 | NA | NA | NA | NA | NA | NA | NA | NA |
| GT-09 | M | NA | NA | NA | NA | NA | NA | NA | NA | NA | NA | NA | NA |
| GT-10^$^ | M | 1 | 3 | 9.4 | 1-2/1-2 | 327 | 76.6 | 9.4 | >280 | 37.3 | 2 | 2 | 2 |
| GT-11 | M | 2 | 2 | 101 | 2-3/NA | 267 | 74.9 | 10 | >238 | 42 | 1 | 2 | 2 |
| GT-12 | M | 2 | NA | 120 | NA | 523 | 74.9 | 10.1 | >289 | 34.6 | 1 | 2 | 2 |
| GT-13 | M | 2 | NA | 161 | UNAFF | 267 | 89.3 | 11.3 | 115 | 28.7 | 1 | 2 | 2 |
| GT-14 | F | NA | NA | 125 | UNAFF | 328 | 83.3 | 11.3 | 116 | 27.4 | 2 | 2 | 2 |
| GT-15 | M | 2 | 1 | 13.6 | 1-2/1-2 | 327 | 81.1 | 7.6 | >298 | 33.2 | 2 | 2 | 2 |
| GT-16 | M | 2 | 3 | 7.3 | 1-2/1-2 | 381 | 52.1 | 7.3 | >300 | 31.1 | 2 | 2 | 2 |
| GT-17 | F | 2 | 3 | 6.4 | 1-2/1-2 | 438 | 44.8 | 14.6 | 272 | 27 | 2 | 2 | 2 |
| GT-18 | F | NA | NA | NA | UNAFF | NA | NA | NA | NA | NA | NA | NA | NA |
| GT-19 | M | NA | NA | NA | UNAFF | NA | NA | NA | NA | NA | NA | NA | NA |
| GT-20 | F | NA | NA | NA | UNAFF | NA | NA | NA | NA | NA | NA | NA | NA |
| GT-21 | M | 2 | 2 | 9.8 | 1-2/1-2 | 281 | 84.4 | 10.7 | >300 | 31.3 | 1 | 2 | 2 |
| GT-22 | F | 3 | 3 | 7 | 1-2/1-2 | 169 | 61.9 | 11.9 | >299 | 31.9 | 2 | 2 | 2 |
| GT-23 | F | 1 | NA | 113 | 1-2/1-2 | 190 | 85.8 | 11.3 | >300 | 37.1 | 1 | 1 | 1 |
| GT-24 | F | 1 | 2 | 10.9 | 1-2/1-2 | 230 | 72.8 | 12.2 | >224 | 32.9 | 2 | 1 | 1 |
| GT-25 | F | 1 | 1 | 13.4 | Prolonged heavy menstruation | 172 | 90.1 | 12.2 | >287 | 35.5 | 1 | 1 | 1 |
| GT-26 | M | 1 | 3 | 6.7 | 1-2/1-2 | 307 | 66.7 | 12 | >300 | 29.6 | 1 | 1 | 1 |
| GT-27 | M | 1 | 2 | 9.8 | 2-3frequent/ NA | 233 | 74.1 | 11.9 | >192 | 36.2 | 2 | 1 | 1 |
| GT-28 | F | 1 | 3 | 5.5 | persistent bleeding, complications | 164 | 64.1 | 19.9 | >223 | 36.3 | 2 | 1 | 1 |
| GT-29 | M | 1 | 2 | 8.8 | 1/1 | 446 | 77.6 | 7.8 | >300 | 35.7 | 2 | 1 | 1 |
| GT-30 | F | 1 | 2 | 9.8 | NA | 389 | 67.8 | 11.8 | >253 | 36.6 | 2 | 1 | 1 |
| GT-31 | F | 1 | NA | 105 | NA | 258 | 80.2 | 10.2 | >267 | NA | 1 | 1 | 1 |
| GT-32 | F | 1 | 2 | 9.8 | NA | 304 | 63.5 | 6.6 | >175 | 36.3 | 1 | 1 | 1 |
| GT-33 | F | 1 | 2 | 9.6 | 1-2/1-2 | 464 | 70.1 | 8.6 | >232 | 32.7 | 1 | 1 | 1 |
| GT-34 | M | NA | NA | NA | 1/1 | NA | NA | NA | NA | NA | NA | NA | NA |
| GT-35 | F | 1 | 3 | 7.8 | 1/1 | 250 | 77.1 | 13.2 | >227 | 26.5 | 1 | 1 | 1 |
| GT-36 | M | 1 | 1 | 9.7 | 1/1 | 398 | 75.5 | 9.8 | >300 | 33.6 | 2 | 1 | 1 |
| GT-37 | F | 1 | 3 | 11.2 | 1/1 | 267 | 78 | 7.8 | >175 | 36.1 | 2 | 1 | 1 |
| GT-38 | M | NA | NA | NA | UNAFF father | NA | NA | NA | NA | NA | NA | NA | NA |
| GT-39 | F | 1 | 2 | 10.4 | 2-3/NA | 280 | 67.7 | 7.9 | >235 | 33.4 | 2 | 1 | 1 |
| GT-40 | M | 1 | 1 | 12 | 2-3/NA | 263 | 78.1 | 8.3 | >228 | 31.3 | 2 | 1 | 1 |
| GT-41 | F | 1 | 3 | 7.7 | 2-3 frequent/ NA | 238 | 62 | 8.8 | NA | 32.6 | 1 | 1 | 1 |
| GT-42 | M | NA | NA | NA | UNAFF father | NA | NA | NA | NA | NA | NA | 2 | 2 |
| GT-43 | M | 1 | 3 | 10.2 | 1/1 | 400 | 76.3 | 10.5 | >228 | 34.9 | 2 | 1 | 1 |
| GT-44 | F | NA | NA | NA | UNAFF | NA | NA | NA | NA | NA | NA | NA | NA |
| GT-45 | M | NA | NA | NA | UNAFF | NA | NA | NA | NA | NA | NA | 2 | 2 |
| GT-46 | F | NA | NA | NA | UNAFF | NA | NA | NA | NA | NA | NA | 2 | 2 |
| GT-47 | F | 1 | 2 | 11.6 | Prolonged heavy menstruation | 284 | 82.3 | 10.4 | >200 | 33.7 | 2 | 1 | 1 |
| GT-48 | M | 1 | 2 | 8.9 | 1/1 | 335 | 87 | 12.5 | >182 | 33.5 | 1 | 1 | 1 |
| GT-49 | F | NA | NA | NA | 1/1 | NA | NA | NA | NA | NA | NA | NA | NA |
| GT-50 | M | NA | NA | NA | 1/1 | NA | NA | NA | NA | NA | NA | NA | NA |
| GT-51 | F | NA | NA | NA | 1/1 | NA | NA | NA | NA | NA | NA | NA | NA |
| GT-52 | M | 1 | 2 | 11.3 | 1/1 | 291 | 80.4 | 10.9 | >217 | 35.5 | 3 | 1 | 1 |
| GT-53 | F | NA | NA | NA | 1/1 | NA | NA | NA | NA | NA | NA | NA | NA |
| GT-55 | M | 1 | 2 | 8.3 | 2-3 frequent/ NA | 182 | 81.4 | 9.4 | >175 | 37.7 | 1 | 1 | 1 |
| GT-56 | F | 1 | 2 | 11.6 | 1/1 | 377 | 78.4 | 7.5 | >278 | 36.7 | 1 | 2 | 2 |
| GT-57 | M | NA | NA | NA | 3/NA | NA | NA | NA | NA | NA | NA | NA | NA |
| GT-58 | F | 1 | 3 | 6.5 | 1/1 | 465 | 63.8 | 8.1 | NA | 32.6 | 1 | 1 | 1 |
| GT-59 | F | NA | NA | NA | UNAFF | NA | NA | NA | NA | NA | NA | NA | NA |
| GT-60 | F | NA | NA | 105 | UNAFF | 258 | 80.2 | 10.2 | NA | NA | NA | NA | NA |
| GT-61 | M | 3 | 2 | 13.5 | 1/1 | 125 | 83.9 | 9.7 | >298 | 29 | 1 | 2 | 2 |
| GT-62 | F | 2 | 3 | 6.2 | 1/1 | 266 | 55 | 8.7 | >257 | 26 | 2 | 2 | 2 |
| GT-63 | F | 1 | 3 | 6.5 | NA | 286 | 59.6 | 10.4 | >234 | 34.4 | 1 | 1 | 1 |
| GT-64 | M | 1 | NA | NA | 1/1 | NA | NA | NA | NA | NA | NA | NA | NA |
| GT-65 | F | 1 | NA | NA | 1/1 | NA | NA | NA | NA | NA | NA | NA | NA |
| GT-66 | F | NA | NA | NA | NA | NA | NA | NA | NA | NA | NA | NA | NA |
| GT-67 | F | NA | NA | NA | NA | NA | NA | NA | NA | NA | NA | NA | NA |
| GT-68 | M | NA | NA | NA | NA | NA | NA | NA | NA | NA | NA | NA | NA |
| GT-69 | M | NA | NA | NA | NA | NA | NA | NA | NA | NA | NA | NA | NA |
| GT-70 | NA | 3 | NA | 10.1 | NA | 380 | 65.6 | 7.9 | NA | 34.5 | NA | NA | NA |
| GT-71 | NA | 3 | NA | 11.3 | NA | 289 | 75.2 | 8.6 | NA | 40.5 | NA | NA | NA |
| GT-72 | NA | 3 | NA | 11.3 | NA | 283 | 76.7 | 9.2 | NA | 39.6 | NA | NA | NA |
| GT-73 | NA | 3 | NA | 15.2 | NA | 411 | 86.5 | 8.6 | 0 | NA | NA | NA | NA |
| GT-74 | NA | 3 | NA | 15.6 | NA | 259 | 85.5 | 9.2 | 0 | 54.8 | NA | NA | NA |
| GT-75 | NA | 3 | NA | 15.3 | NA | 274 | 86.4 | 8.9 | NA | 47.4 | NA | NA | NA |
| GT-76 | M | 1 | NA | NA | 1/1 | NA | NA | NA | NA | NA | NA | 1 | 1 |

^*^GT Type-I (1) /Type-II (2) /Variant (3)

^#^Bleeding severity: Mild (1)/ Moderate (2)/ Severe (3)

^$^No DNA available for testing

^^^ Negative (1)/ Positive (2)

NA: Not available, UNAFF: unaffected, PFA: Platelet Function Assay

**Supplementary Table 4: variants frequencies in different databases**

| Gene | Exon | Variant | ExAC/  1000 genomes | SHGP  (3206 exomes) | rs or HGMD |
| --- | --- | --- | --- | --- | --- |
| *ITGB3* | exon 4 | c.437T>C:p.L146P | 0/0 | 0/0 | No HGMD |
| *ITGB3* | exon 5 | c.727G>C:p.D243H | 0/0 | 0 | CM931192 |
| *ITGB3* | exon 5 | c.662C>T:p.T221M | 3.295e-05/0 | 0 | CM086336 |
| *ITGB3* | exon 7 | c.985A>G:p.N329D | 0.0001/0 | 0.007 | rs201550717 |
| *ITGB3* | exon 10 | c.1265G>A:p.S422N | 0/0 | 0.0015 | rs372644002/CM061072 |
| *ITGB3* | exon 10 | c.1539delC:p.S513fs | 0/0 | 0 | No HGMD |
| *ITGB3* | exon 11 | c.1835G>A:p.C612Y | 0/0 | 0 | No HGMD |
| *ITGB3* | exon 13 | c.2112delC:p.I704fs | 0/0 | 0.0155 | No HGMD |
| *ITGB3* | exon 14 | c.2301+9C>G | 0/0 | 0.004 | No HGMD |
| *ITGB3* | exon 15 | c.2302-1G>A | 0/0 | 0.0001 | No HGMD |
| *ITGA2* | exon 2 | c.185+8T>C | 0/0 | 0 | No HGMD |
| *ITGA2* | exon 6 | c.630+10A>G | 0.7213/0.702 | 0.57 | rs1421933 |
| *ITGA2* | exon 8 | c.780-11A>T | 0.0002/0.0005 | 0.024 | rs202058704 |
| *ITGA2* | exon 9 | c.958C>G:p.L320V | 0/0 | 0 | No HGMD |
| *ITGA2* | exon 9 | c.967delA:p.K323fs | 0/0 | 0 | No HGMD |
| *ITGA2* | exon 13 | c.1535G>A:p.G512D | 0/0 | 0.0003 | No HGMD |
| *ITGA2* | exon 14 | c.1650A>C:p.E550D | 0/0 | 0.0006 | No HGMD |
| *ITGA2* | exon 21 | c.2572-7T>C | 3.331e-05/0 | 0.0003 | rs375862598 |
| *ITGA2B* | exon 4 | c.558C>G:p.Y186X | 0/0 | 0 | CD098124 |
| *ITGA2B* | exon 5 | c.574+3A>T | 0/0 | 0.0006 | No HGMD |
| *ITGA2B* | exon 5 | c.574+5G>A | 0/0 | 0 | CS119984 |
| *ITGA2B* | exon 5 | c.574+6C>G | 0/0 | 0.0003 | No HGMD |
| *ITGA2B* | exon 6 | c.575-10T>C | 0/0 | 0.0012 | No HGMD |
| *ITGA2B* | exon 8 | c.800-7T>C | 0/0 | 0.0059 | No HGMD |
| *ITGA2B* | exon 11 | c.985G>T:p.V329F | 0/0 | 0 | CM030472 |
| *ITGA2B* | exon 12 | c.1142C>T:p.T381I | 0/0 | 0 | No HGMD |
| *ITGA2B* | exon 13 | c.1210+5G>A | 0/0 | 0^*^ | No HGMD |
| *ITGA2B* | exon 17 | c.1616T>G:p.L539R | 0/0 | 0 | CD052111 |
| *ITGA2B* | exon 17 | c.1620G>C:p.Q540H | 0/0 | 0 | No HGMD |
| *ITGA2B* | exon 17 | c.1651C>T:p.R551W | 0/0 | 0 | CM066102/CM093510 |
| *ITGA2B* | exon 19 | c.1879-2A>G | 0/0 | 0.002 | rs77229108/CS099779 |
| *ITGA2B* | exon 20 | c.1947-9T>C | 0/0 | 0.0006 | No HGMD |
| *ITGA2B* | exon 26 | c.2613delG:p.G871fs | 0/0 | 0.015 | No HGMD |

**Supplementary Table 5: Tertiary Analysis- Pathogenicity prediction for unreported variants**

| Gene | Variant | ExAC/  1000 genome | SHGP | **CADD** | | **PredictSNP2** | | | | | | Variant ranker score |
| --- | --- | --- | --- | --- | --- | --- | --- | --- | --- | --- | --- | --- |
|  |  |  |  | Raw  Score | PHRED | Predict  SNP2 | CADD | DANN | FATHMM | FunSeq2 | GWAVA |  |
| *ITGA2* | NM_002203:exon9:c.958C>G:p.L320V | 0/0 | 0 | 5.06533 | 25.3 | 87% | 67% | 66% | 56% | 62% | NA | 15.83 |
|  | NM_002203:exon9:c.967delA:p.K323fs | 0/0 | 0 | 5.049065 | 25.2 | NA | NA | NA | NA | NA | NA | NA |
|  | NM_002203:exon14:c.1650A>C:p.E550D | 0/0 | 0.0006 | 4.155255 | 23.8 | 87% | 56% | 62% | 82% | 62% | NA | 14.833 |
|  | NM_002203:exon13:c.1535G>A:p.G512D | 0/0 | 0.0003 | 6.677305 | 32 | 87% | 84% | 71% | 83% | 62% | NA | 18.33 |
| *ITGA2B* | NM_000419:exon13:c.1210+5G>A | 0/0 | 0 | 2.541594 | 19.73 | 97% | 96% | 99% | 98% | 74% | 82% | 6 |
|  | NM_000419:exon8:c.800-7T>C | 0/0 | 0.0059 | 1.14813 | 11.47 | 91% | 69% | 68% | NA | NA | 53% | 5 |
|  | NM_000419:exon12:c.1142C>T:p.T381I | 0/0 | 0 | 5.329677 | 25.8 | 87% | 63% | 72% | 63% | 62% | NA | 17.33 |
|  | NM_000419:exon17:c.1620G>C:p.Q540H | 0/0 | 0 | 5.056391 | 25.2 | 87% | 71% | 66% | 79% | 62% | NA | 17.33 |
|  | NM_000419:exon17:c.1616T>G:p.L539R | 0/0 | 0 | 5.465117 | 26.2 | 87% | 77% | 68% | 83% | 62% | NA | 17.33 |
| *ITGB3* | NM_000212:exon14:c.2301+9C>G | 0/0 | 0.004 | 5.973231 | 27.7 | 87% | 84% | 77% | 80% | 62% | NA | 21.33 |
|  | NM_000212:exon11:c.1835G>A:p.C612Y | 0/0 | 0 | 6.431147 | 29.8 | 87% | 52% | 70% | 83% | 62% | 51% | 21.33 |
|  | NM_000212:exon4:c.437T>C:p.L146P | 0/0 | 0 | 5.973231 | 27.7 | 87% | 84% | 77% | 80% | 62% | NA | 21.33 |

NA: Not available

**References**

1. Nurden, A. T., Pillois, X. & Nurden, P. Understanding the genetic basis of Glanzmann thrombasthenia: implications for treatment. Expert review of hematology 5, 487-503, doi:10.1586/ehm.12.46 (2012).

2. Marchler-Bauer, A. et al. CDD/SPARCLE: functional classification of proteins via subfamily domain architectures. Nucleic acids research 45, D200-d203, doi:10.1093/nar/gkw1129 (2017).

3. Marchler-Bauer, A. & Bryant, S. H. CD-Search: protein domain annotations on the fly. Nucleic acids research 32, W327-331, doi:10.1093/nar/gkh454 (2004).

4. Marchler-Bauer, A. et al. CDD: NCBI's conserved domain database. Nucleic acids research 43, D222-226, doi:10.1093/nar/gku1221 (2015).

5. Marchler-Bauer, A. et al. CDD: a Conserved Domain Database for the functional annotation of proteins. Nucleic acids research 39, D225-229, doi:10.1093/nar/gkq1189 (2011).
